# Neutrophil Metabolic Shift during Their Lifecycle: Impact on Their Survival and Activation

**DOI:** 10.3390/ijms21010287

**Published:** 2019-12-31

**Authors:** Louise Injarabian, Anne Devin, Stéphane Ransac, Benoit S. Marteyn

**Affiliations:** 1 Université de Strasbourg, Institut de Biologie Moléculaire et Cellulaire, CNRS, Architecture et Réactivité de l’ARN, UPR9002, F-67000 Strasbourg, France; l.injarabian@ibmc-cnrs.unistra.fr; 2 Université de Bordeaux, IBGC, UMR 5095, 1 rue Camille Saint Saëns, 33077 Bordeaux Cedex, France; anne.devin@ibgc.cnrs.fr (A.D.); ransac@ibgc.cnrs.fr (S.R.); 3 Institut Pasteur, Unité de Pathogenèse des Infections Vasculaires, 75724 Paris, France

**Keywords:** neutrophils, energetic metabolism, infection, inflammation, nutrient availability, oxygen sensing

## Abstract

Polymorphonuclear neutrophils (PMNs) are innate immune cells, which represent 50% to 70% of the total circulating leukocytes. How PMNs adapt to various microenvironments encountered during their life cycle, from the bone marrow, to the blood plasma fraction, and to inflamed or infected tissues remains largely unexplored. Metabolic shifts have been reported in other immune cells such as macrophages or lymphocytes, in response to local changes in their microenvironment, and in association with a modulation of their pro-inflammatory or anti-inflammatory functions. The potential contribution of metabolic shifts in the modulation of neutrophil activation or survival is anticipated even though it is not yet fully described. If neutrophils are considered to be mainly glycolytic, the relative importance of alternative metabolic pathways, such as the pentose phosphate pathway, glutaminolysis, or the mitochondrial oxidative metabolism, has not been fully considered during activation. This statement may be explained by the lack of knowledge regarding the local availability of key metabolites such as glucose, glutamine, and substrates, such as oxygen from the bone marrow to inflamed tissues. As highlighted in this review, the link between specific metabolic pathways and neutrophil activation has been outlined in many reports. However, the impact of neutrophil activation on metabolic shifts’ induction has not yet been explored. Beyond its importance in neutrophil survival capacity in response to available metabolites, metabolic shifts may also contribute to neutrophil population heterogeneity reported in cancer (tumor-associated neutrophil) or auto-immune diseases (Low/High Density Neutrophils). This represents an active field of research. In conclusion, the characterization of neutrophil metabolic shifts is an emerging field that may provide important knowledge on neutrophil physiology and activation modulation. The related question of microenvironmental changes occurring during inflammation, to which neutrophils will respond to, will have to be addressed to fully appreciate the importance of neutrophil metabolic shifts in inflammatory diseases.

## 1. Introduction

**Figure 1:**
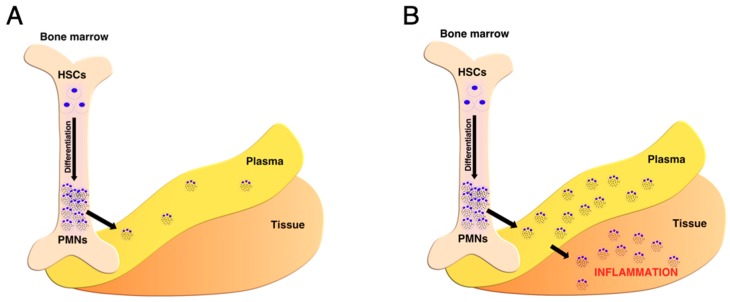
Neutrophil presence in the bone marrow, plasma and tissues under physiological (**A**) and under pathophysiological conditions (**B**). PMNs—polymorphonuclear neutrophils; HSCs—hematopoietic stem cells.

Polymorphonuclear neutrophils (neutrophils, PMNs) like all migratory cells face various microenvironments during their lifecycle from their site of production to their site of action or clearance (view Figure 1). Neutrophils are the most abundant leukocytes in the circulation, which represent 50% to 70% of circulating leukocytes. Circulating neutrophils are fully differentiated cells and are believed to have a relatively short lifespan, which is likely largely dependent on environmental parameters. Bone marrow and blood plasma homeostasis are expected to play a central role in maintaining neutrophils in a quiescent state under basal conditions, even though the parameters involved in this process remain largely undefined. In eukaryotic cells, energy is produced by glycolysis in the presence of glucose, mitochondrial respiration (oxidative phosphorylation) in the presence of oxygen, and the tricarboxylic acid (TCA) cycle, in the presence of oxygen and pyruvate, glutamine, or free fatty acids. Under a basal condition, neutrophils are mainly glycolytic and contain few mitochondria [1]. However, the contribution of mitochondrial respiration or the TCA cycle to neutrophil energy production has not been fully investigated upon activation. We have demonstrated that oxygen exposure has a deleterious effect on neutrophil viability [2,3,4], even though the potential involvement of mitochondria in this process has not been established. As a consequence, the respective abundance of metabolites and substrates, such as glucose, glutamine, and oxygen in the neutrophil microenvironment appears to be critical for neutrophil physiology, survival, and activation. 

This point may be illustrated by the metabolic shift occurring during neutrophil differentiation (granulopoiesis) from hematopoietic stem cells (HSCs) in the bone marrow. The importance of metabolic shifts in immune cell adaptation to their microenvironment has been extensively demonstrated in the polarization of tumor-associated macrophages (TAM), which allows the characterization of two populations: M1 and M2. M1 macrophages are mainly glycolytic with limited oxygen consumption capacity. In comparison, M2 macrophages mainly use oxidative phosphorylation for energy production. The M1/M2 metabolic shift has a functional correlation, where M1 exhibits a pro-inflammatory and anti-tumoral phenotype and M2 exhibits an anti-inflammatory and pro-tumoral one [5,6]. Similar classification of tumor-associated neutrophil (TAN) populations has been proposed by Fridlender (namely N1/N2 populations) in response to TGF-β [7]. This concept is currently evaluated in different inflammatory models and is being discussed [8]. Although no metabolic shifts or differences have yet been reported between the N1 and N2 populations, their identification strongly suggests that neutrophils can efficiently respond and adapt to local microenvironmental changes.

The ability of neutrophils to sense and adapt to changes of microenvironmental parameters, such as the pO_2_ or the glucose concentration, is well documented through transcriptional regulations (e.g., HIF1-dependent and HIF2-dependent) [9]. However, the impact of neutrophil metabolic activity or activation on its microenvironment is likely underestimated and will be further discussed. In addition, it remains unclear whether heterogeneous microenvironments encountered by neutrophils during their lifecycle modulate and shape the population homogeneity (N1/N2 or high-density/low-density neutrophils) if these processes are reversible or not.

In this case, current knowledge on neutrophil metabolism under basal conditions will be reviewed, and potential metabolic shifts occurring within inflamed tissues or upon cell activation will be described. We aim to elucidate how neutrophils adapt to stressful conditions encountered during their lifecycle, by either tuning or modulating their metabolic pathways or using stored metabolites.

## 2. Available Metabolites in the Bone Marrow, Plasma, and Inflamed Tissues

Under basal conditions, blood plasma homeostasis is expected to limit neutrophil activation and to sustain their viability. Conversely, the inflammatory tissue microenvironment allows neutrophil activation (migration, degranulation, phagocytosis, or reactive oxygen species (ROS) production). The relative abundance of glucose, glutamine, and oxygen in plasma and in inflammatory tissues is reviewed hereafter (view Table 1).

### 2.1. Bone Marrow Homeostasis

The bone marrow microenvironment plays a crucial role in the control of hematopoietic stem cell proliferation and differentiation [10]. However, to our knowledge, the concentrations of glucose and glutamine in this microenvironment remain unknown. Further investigations are required to determine these key parameters to better appreciate and understand the physiology of mature neutrophils stored in the bone marrow, which represent the vast majority of the neutrophil population. The bone marrow has long been described as a hypoxic environment, despite being densely perfused. This perception has been recently confirmed by local pO_2_ quantification in mice bone marrow by Spencer and colleagues using two-photon phosphorescence lifetime microscopy, which is a non-invasive and non-destructive quantification method [11]. In this study, the authors demonstrated that the bone marrow oxygenation level is heterogeneous but still rather low in all compartments (below 32 mmHg, or 4.2%). Surprisingly, the most hypoxic environment was measured in peri-sinusoidal regions (9.9 mmHg, or 1.3%), while the endosteal region was less hypoxic (21.9 mmHg, or 2.9%), which is being perfused by small arteries.

### 2.2. Plasma Homeostasis

Under basal conditions, the glucose level remains tightly controlled at 5.6–6.9 mM in the blood. These values are relatively high with regard to neutrophil glucose metabolic needs and to the maintenance of their viability. Hyperglycemia has been reported to have an adverse effect on neutrophil activation (e.g., mobilization defect) [12,13], induction of neutrophil extracellular traps (NET) formation [14], and inflammasome activation [15], even though no direct link with potential metabolic shifts has been reported. To our knowledge, the neutrophil metabolic adaptation under hypoglycemia has not been investigated.

Similar to glucose, l-glutamine is well maintained in the plasma at a 0.4–0.6 mM concentration. Being the most abundant amino acid in the body, it fuels the production of the most abundant intracellular amino acid l-glutamate [16]. A constant glutaminemia is crucial for proper functioning of the immune system. Compared to hyperglycemia or hypoglycemia, neither changes in glutaminemia in plasma, nor its impact on a potential neutrophil metabolic shift have yet been investigated. 

The plasma oxygen level is contained at very low concentrations, as described by Pittman [17]. Since 98% of oxygen is transported by red blood cells, the dissolved fraction represents only 2%. Plasma oxygenation has never been quantified in situ due to technical and ethical limitations. Based on a theoretical calculation of plasma oxygenation (Henry’s law of gas diffusion), it is estimated that plasma pO_2_ varies between 1.4 mmHg in veins and 2 mmHg in arteries (with pO_2 vein_=70 mmHg, and pO_2 artery_ = 100 mmHg). Taken these values together with the high amount of glucose in plasma, it is anticipated that the glucose metabolism will be favored for the oxidative metabolism in circulating neutrophils (see below).

pH in the plasma is strictly maintained between 7.35 and 7.45 via large amounts of carbonic acid and bicarbonate.

### 2.3. Tissue Homeostasis

Glucose passively diffuses from the blood plasma to organs to ensure that the supply meets the needs under basal conditions. Apart from getting nutritional glucose, some organs, such as the liver and muscles, can store glucose in the form of glycogen (100 g and 400 g, respectively). Circulating neutrophils are known to rely on glycolysis to maintain their viability and function [1,18,19]. Glycogen bodies have been observed in quiescent neutrophils (electron microscopy) [20,21] as well as the presence and expression of glycogen synthase (Gly1), which is necessary for glycogen synthesis [22]. The intracellular glycogen concentration in quiescent neutrophils is estimated at 1 µg/1.10^6^ cells [22].

Glutamine is a non-essential amino acid. However, it may be considered conditionally essential during illness since intestines and the immune system utilize a large amount of glutamine during inflammation, which exceeds endogenous production. The glutamine concentration in tissues under basal conditions is relatively poorly known and is estimated to be higher than in the plasma fraction (intracellular concentration 2–20 mM). For example, the measurements performed in human muscles indicated a glutamine concentration ranging from 2–4 mmol/L [23]. In rat hepatocytes, intracellular glutamine was even higher, measured at 7 mM [24]. In mice, basal glutamine concentration in the plasma is around 0.2 mM in organs such as the liver and the kidney at 2.8 and 0.5 µmol/g, respectively [25].

The importance of glutamine concentration during inflammation remains elusive and will be discussed in the next chapter.

The basal level of oxygen in tissues is organ-specific and depends on the local supply and metabolic needs. Physiological O_2_ concentration has been defined as “physioxia” [26,27] and ranges from 1% to 11% in human body compartments [28,29].

Similar to oxygen, pH values tend to vary between different tissue types, whereas skeletal muscle tissue’s pH is 6.8–7.1, colon tissue’s pH is 7.9–8.5, and small intestine tissue’s pH is 7.2–7.5.

### 2.4. Available Metabolites in the Inflamed Tissue Microenvironment

Not much is known about changes in the glucose concentration and local distribution in tissues during sterile inflammation. However, during infection, some intracellular pathogens, such as *Salmonella* and *Brucella* consume glucose, which alters its homeostasis in tissues [30,31]. In the case of *Brucella* infection, a two-fold decrease of intracellular glucose was measured in murine macrophages (from 1 to 0.5 µM, respectively) [31], and reduces the glucose supply during *Listeria monocytogenes* infection, which increases the mice survival rate [32].

Many studies report the benefit of glutamine supplementation on intestinal inflammation. In general, glutamine sustains enterocyte proliferation, suppresses pro-inflammatory pathways, and protects host cells from apoptosis, among other functions [33]. Under continuous stress conditions, muscles and lungs are known to release glutamine, since other organs increase glutamine uptake and consumption. In human tissues, the intracellular glutamine concentration decreases compared to murine tissues where a two-fold increase in glutamine concentration was observed in plasma and tissues samples during stress conditions. Measurements done in patients with chronic inflammatory stress, causing intestinal permeability, showed a correlation between the severity of inflammation and decreased glutamine levels in human intestines (mucosa) and plasma [34]. In this particular study, tissue glutamine concentration of inflammatory mucosa ranged from 1.4 to 4 mM, whereas higher values were measured in patients with low inflammation markers [34]. A decrease of glutamine concentration was associated with inflammatory activity rather than nutritional depletion, which suggests an increase in local glutamine consumption. Values of glutamine fluctuations in case of infection are not well known. However, it was recently shown that *Escherichia coli* can use glutamine during infection, which mediates their protection from acid stress and copper ion toxicity, which lowers the local glutamine concentration [35].

It is well accepted that the local oxygen level decreases during inflammation, as first demonstrated in a colitis mouse model by Karhausen and colleagues [36]. This observation has been further confirmed by other groups, which leads to the concept of “inflammatory hypoxia” [37]. Recently, our group demonstrated a severe hypoxia induction during *Shigella* infection in the guinea pig colon, which refers to the “infectious hypoxia” concept [38]. The main cause of hypoxia induction is the consumption of oxygen by the bacteria aerobic respiration [39]. Similar observations have been seen during *Staphylococcus aureus* [40] (reviewed by Hajdamowicz et al. [41]) and *Salmonella typhimurium* infections [42] or upon *Mycobacterium tuberculosis* granuloma formation [43].

Similar to pathogens, tumor cells are capable of shaping the local microenvironment to promote their growth and survival.

Cancer cells are usually characterized by a high glycolytic phenotype, which produces lactate and lowers the extracellular pH in the tumor microenvironment (TME). However, tumors can have a glycolytic (mainly) or an oxidative metabolism. TMEs of each phenotype differ in nutrient availability. Some parameters are similar in both, such as hypoxia (<2%) and acidosis (pH 6.5–6.9) [44], which are considered potential cancer drug targets (reviewed by Bailey et al. [45]). The glycolytic tumor cell metabolism is highly impacted by the stabilization of the hypoxia-inducing factor (HIF) pathway, which upregulates glycolysis. Glycolytic tumors have a higher glucose consumption rate, compared to other cells present in the TME [46]. Therefore, glycolytic TMEs are characterized by low glucose availability, high lactate concentration, and high acidity. On the other hand, oxidative TMEs are characterized by low fatty acid, amino acid, and oxygen availability (reviewed by Scharping and Delgoffe [47]).

In addition to consuming most of the glucose in the TME, glycolytic tumor cells were shown to have an increased glutamine metabolism. It is, therefore, not surprising that glutamine levels in the TME are low [48,49,50]. However, a recent publication challenged the importance of glutamine in tumor metabolism by demonstrating that glutamine consumption was not increased and remained relatively low in in vivo mouse lung cancer models [51]. These opposing findings finely illustrate the heterogeneity of tumor cell metabolism and potential biases occurring during in vitro experiments. 

Tumor hypoxia is perceived as a deleterious factor in cancer therapy. Hypoxia within the tumor varies and creates a gradient of 0.1–6% O_2_, depending on the size and the vascularization of the tumor [52]. Despite the fact that hypoxia induction has been reported in various inflammatory models, no pO_2_ quantification has yet been achieved in situ with non-disruptive quantification methods. This likely represents one of the most challenging goals in the field.

pH values during inflammation tend to differ dramatically from homeostatic values. Inflammation sites are characterized by a drop in pH rather than an increase. The acidification is linked to the glycolytic metabolism of infiltrating immune cells in case of infection and to the glycolytic metabolism of cancer cells in tumor microenvironments, which produces and releases lactic acid.

## 3. Neutrophil Metabolism

**Figure 2:**
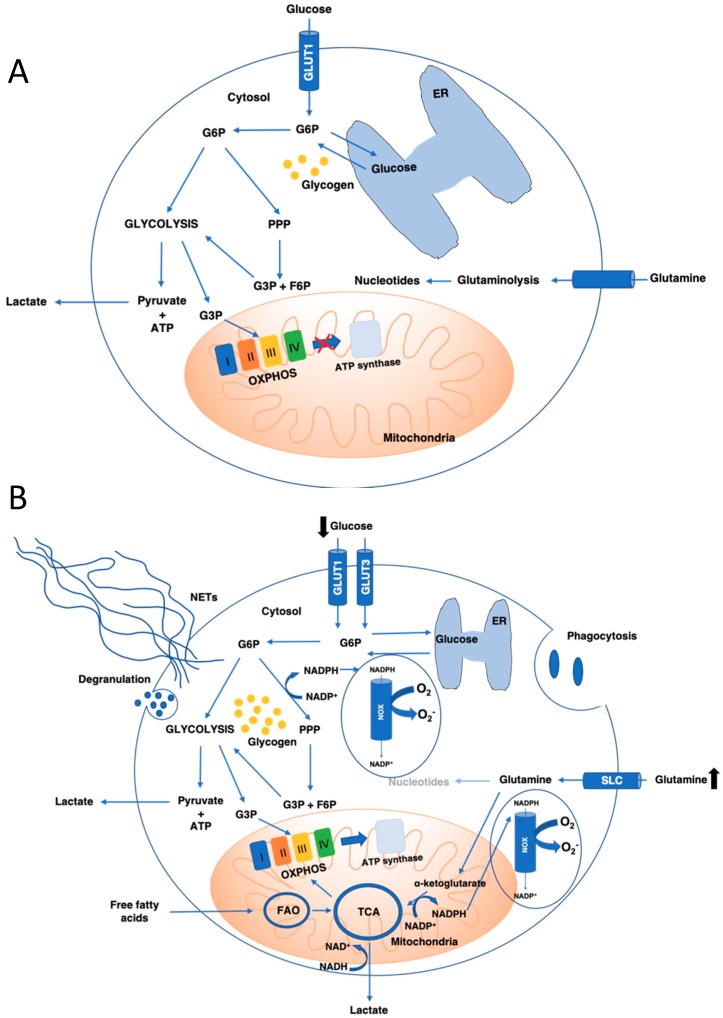
Metabolic pathways in non-activated (**A**) and activated neutrophils (**B**). G6P—Glucose-6-phosphate; PPP—Pentose Phosphate Pathway; GLUT1—Glucose Transporter 1; SLC—Solute Carrier; OXPHOS—Oxidative Phosphorylation; G3P—Glycerol-3-Phosphate; F6P—Fructose-6-Phosphate; FAO—Fatty Acid Oxidation; TCA—Tricarboxylic Acid Cycle; NOX—NADPH Oxidase; ER—Endoplasmic Reticulum; NETs—Neutrophil Extracellular Traps.

The most thoroughly described metabolism in neutrophils is glycolysis since circulating neutrophils are considered to be highly glycolytic under physiological conditions (view Figure 2). However, additional metabolic pathways have been described in neutrophils, such as the pentose phosphate pathway (PPP), The Krebs/Tricarboxylic (TCA) cycle, oxidative phosphorylation (OXPHOS), and a fatty acid oxidation (FAO) pathway (view Figure 2).

### 3.1. Glucose Metabolism: Glycolysis and Pentose Phosphate Pathways

Neutrophils rely on intracellular glucose shuffling to fuel their glucose-dependent metabolisms: glycolysis and PPP.

Glucose shuffling per se consists in G6P/glucose cycling through the G6P transporter (G6PT)/G6Pase-beta complex. Shortly, G6P is imported into the endoplasmic reticulum (ER) via the G6PT, where it is hydrolyzed back into glucose by the G6Pase-β. Glucose can return to the cytoplasm where it is immediately converted back into G6P. This pathway, which limits available G6P in the cytoplasm, ensures tight control over the glucose metabolism fluxes. 

Defects in neutrophil glucose cycling result in decreased glucose uptake and lower intracellular G6P, but also impaired energy metabolism [53,54,55,56].

Several pathologies linked to defects in G6P/glucose cycling have been described, such as the glycogen storage disease type Ib (GSDIb, deficiency in G6PT) and severe congenital neutropenia syndrome (SCN4, deficiency in G6Pase-β). Patients with these pathologies suffer from neutropenia and neutrophil dysfunctions due to glycolysis inhibition [57], which highlights the importance of the glucose metabolism for neutrophil survival and functioning.

Glycolysis is a ubiquitous energy metabolism, which does not differ between cell types. Extracellular glucose is imported into human neutrophils via glucose transporter 1 (GLUT1), which is expressed basally and upregulated in glucose-rich environments [57]. Other glucose transporters, such as 3 and 4, are also expressed but poorly described in neutrophils. If, in humans, upon activation with phorbol 12-myristate 13-acetate (PMA), only glucose transporter (GLUT) 1 and GLUT3 are upregulated [58,59], then in rats, increased GLUT4 expression has also been described when activated by the platelet activation factor [60]. Upon uptake, glucose is immediately transformed into glucose-6-phosphate (G6P) by the hexokinase, where localization may be modulated by the neutrophil activation status [61]. The consumption of each G6P molecule will lead to the production of two molecules of pyruvate, two molecules of ATP, and two molecules of NADH. In aerobic conditions, pyruvate can be oxidized in mitochondria through the TCA cycle (see below). However, in neutrophils, pyruvate is converted into lactate, which enables the regeneration of NAD+ essential for the continuity of glycolysis [1,18,62].

Another glucose-dependent pathway in neutrophils is the PPP, which is also known as the hexose monophosphate shunt, that has been studied in quiescent neutrophils and during activation (NET formation) [63]. The PPP encompasses an oxidative and a non-oxidative phase. During the oxidative phase, G6P-dehydrogenase (G6PD), 6-phosphogluconolactonase, and 6-phosphogluconate dehydrogenase convert G6P into CO_2_, ribulose-5-phosphate, and nicotinamide adenine dinucleotide phosphate (NADPH). NADPH production is essential for the maintenance of the redox balance under stress situations. During the non-oxidative phase, several enzymes will be involved: the ribose-5-phosphate isomerase, the ribulose-5-phosphate 3-epimerase, a transketolase, and a transaldolase, which leads to the conversion of ribulose-5-phosphate into nucleic acids, sugar phosphate precursors, or glycolytic precursors, such as fructose-6-phosphate (F6P) and glyceraldehyde-3-phosphate (G3P). Thus, PPP and glycolysis share a pool of G3P and F6P yielding in lactate or pyruvate.

In neutrophils, PPP-dependent NADPH production was shown to be essential for the cytosolic NADPH oxidase (NOX)-dependent ROS production for NET induction [63]. In addition, inhibition of the PPP key enzyme, G6PD, in high glucose concentrations, was also shown to reduce superoxide production [64]. In leukocytes, NADPH produced via the PPP is essential for catalase positive bacteria killing [65]. Cooper et al. reported in an old case-study that neutrophils from a patient lacking G6PD with a functional NOX had deficient bactericidal functions [65]. However, further investigations are required to fully appreciate the contribution of PPP to neutrophil survival and antimicrobial activity.

Even if neutrophils are known to rely mainly on glycolysis, the glycolytic flux is often measured by the consumption of glucose and production of lactate. However, as seen previously, glucose consumed by the PPP can equally yield lactate production. Therefore, the importance of glycolysis has potentially been overestimated, especially during neutrophil activation.

### 3.2. Glutamine Metabolism

Under physiological conditions, glutamine is used to produce precursor nucleotides for RNA and DNA synthesis. The metabolization of glutamine results in glutamate, aspartate, lactate, and ammonia production.

Nevertheless, under pathophysiological conditions, when glucose supply is limited, cells, including neutrophils, can switch to the utilization of glutamine to meet their energetic need [16,66,67,68,69]. In short, glutamine can enter the cell via several solute carrier type transporters (SLCs), such as the sodium-coupled neutral amino acid transporter (SNAT) family proteins. After entering the cytosol of neutrophils (and macrophages), glutamine is not fully oxidized and is converted into glutamate. Glutamate enters the mitochondria and is converted to α-ketoglutarate, which oxygenates NAD^+^ into NADH. α-ketoglutarate can enter the TCA cycle (see below) and produce malate, which is then converted to pyruvate via the malate dehydrogenase, which oxygenates NADP^+^. Pyruvate is then converted into lactate in the cytosol or contributes to the oxidative metabolism (OXPHOS) in the mitochondria. However, in low oxygen conditions (characteristic to inflammation), NAD^+^ can be regenerated through the production of lactate. Yet, how cytosolic NAD^+^ gets into mitochondria is not well established, especially since mammalian mitochondria do not synthesize NAD^+^ and are considered impermeable to pyridine nucleotides. It was recently demonstrated that cytosolic NAD^+^ or NADH can be directly transported into mammalian mitochondria. Yet, the transport mechanism as the transporter itself remains unknown [70]. 

Interestingly, in neutrophils, glutamine can be used in higher rates than glucose [71]. Similar to PPP, glutaminolysis plays a role in the production of NADPH and the expression of the NOX complex [72]. In fact, in differentiated cells, such as macrophages and neutrophils, glutamine mainly plays a role during activation. It has been demonstrated that the glutamine consumption rate is highly increased under catabolic conditions.

### 3.3. Mitochondrial Metabolism: TCA Cycle, OXPHOS, and Fatty Acid Oxidation

Mitochondria are involved in many metabolic and cellular functions, such as cell death (apoptosis, pyroptosis), calcium and iron homeostasis, heme biosynthesis, and energy production. As discussed previously, under basal conditions, mitochondria do not contribute significantly to neutrophil energy metabolism and participate only in the initiation of apoptosis [1]. However, recent findings suggest a potential metabolic shift during activation due to changes in the microenvironment, which favor an oxidative metabolism and are described in many neutrophil subpopulations (discussed in Section 5).

ATP is generated via three major pathways: glycolysis, the tricarboxylic acid (TCA) cycle, and oxidative phosphorylation (OXPHOS). 

The TCA cycle produces OXPHOS intermediates from acetyl-CoA oxidation, which are derived from sugars, fats, or amino acids. OXPHOS produces ATP via a series of oxidation-reduction reactions, which creates a membrane electrochemical potential (∆Ψ_m_). The ∆Ψ_m_ is generated through the coupling of electron transfer and H^+^ pumping via four complexes (C) in the mitochondrial inner membrane. These complexes are CI (NADH- ubiquinone oxidoreductase), CII (succinate-ubiquinone oxidoreductase), CIII (ubiquinol-cytochrome c oxidoreductase), and CIV (cytochrome c oxidase). Complex I, III, and IV are the proton pumps, which transfer protons out of the mitochondrial matrix and generates ∆Ψ_m_. Although controversial, it has been shown that the mitochondrial respiratory chain complexes can form supercomplexes [73], containing several copies of CI, CIII, and CIV within one respiratory chain. The association of four or more copies of CIV was shown to enhance significantly the efficiency of CI and CIII to transfer electrons [74,75], which creates an increased membrane potential and produces more ATP. It was suggested that the lack of supercomplexes in circulating neutrophils may be the cause of a defective OXPHOS contribution to energy production under physiological conditions [61]. In neutrophils, ∆Ψ_m_ was shown to be mainly maintained via the transfer of electron from glycolysis to CIII, via the glycerol-3-phosphate (G3P) shuttle [62]. G3P is a product of glucose metabolism that can enter mitochondria where it is re-oxidized on the outer surface of the inner mitochondrial membrane. Although, in most cells, a membrane potential is coupled to ATP synthesis, it seems to differ in circulating neutrophils [62]. 

Fatty acid oxidation (FAO) is essential for the production of several enzymes, hormones, and cell membrane components. In the cytosol, fatty acids are converted by the acyl-CoA synthetases into fatty acyl-CoA esters, which enter the mitochondria for subsequent oxidation. FAO consists of four enzymatic reactions: dehydrogenation, hydration, another dehydrogenation, and thiolysis, which results in one acetyl-CoA molecule, NADH, H^+^, FADH_2_, and a fatty acyl-CoA ester. After the first reaction, the fatty acyl-CoA ester is shortened by two carbon atoms and can return to the FAO pathway, where it will be re-oxidized until only two acetyl-CoA molecules remain. Acetyl-CoA then enters the mitochondrial TCA cycle where it will be oxidized into CO_2_ and H_2_O, generating additional FADH_2_ and NADH, H^+^. Electrons from both beta-oxidation and the TCA cycle can then be used by the OXPHOS system to generate ATP. 

To sum up, the role of mitochondrial oxidative metabolism in the function and activation of neutrophils has become more relevant in recent years due to the discovery of heterogenous neutrophil populations. Some neutrophil key functions, which were difficult to study before, have now been revealed to depend on mitochondrial functions [76]. For example, neutrophil chemotaxis is impaired when neutrophils lack a functional membrane potential or ATP synthase [77], which indicates the importance of mitochondria and mitochondrial metabolism during neutrophil transmigration into tissues.

## 4. Changes in Metabolism

### 4.1. Metabolic Shift from Hematopoietic Stem Cells to Mature Neutrophils

Hematopoietic stem cells (HSCs, CD34+) residing in the hypoxic niche of the bone marrow [11,78] remain in a resting quiescent state [79,80], exhibiting low bioenergetic activity [80,81,82]. HSCs glycolytic metabolism is directly linked to the stability of HIF1α, which is an oxygen-sensitive transcription factor, mainly involved in the expression of glycolytic enzyme genes. HSCs can exit the quiescent state for the purpose of self-renewal and differentiation, which leads to their asymmetric division, associated with a differential mitochondrial abundance [83]. The daughter cell with a higher mitochondrial content will commit to differentiation and give rise to the blood cell lineages (such as neutrophils), whereas the cell with lower mitochondrial content will re-enter the quiescent phase [83,84,85,86,87]. The ability of HSCs to maintain a low mitochondrial pool is now considered a hallmark of stemness reviewed by Papa et al. [88]. It is suggested that the importance of a differential mitochondrial pool between self-renewing and differentiating cells is linked to the control of ROS production [88]. A higher mitochondrial pool will lead to a higher energy yield but also an increased ROS production, both necessary for differentiation. HSCs, on the other hand, are sensitive to oxidative stress and show low endogenous ROS levels [89,90,91,92,93,94]. Moreover, compared to other cell types in the bone marrow, HSCs have an increased glycolytic capacity [80], strongly related to their adaptation to the hypoxic niche of the bone marrow. 

Under basal conditions, neutrophil differentiation from HSCs (granulopoiesis) leads to the sequential formation of myeloblasts (MBs), promyelocytes (PMs), myelocytes (MCs), metamyelocytes (MMs), band cells (BCs), segmented cells (SCs), and mature neutrophils (PMNs). As previously mentioned, HSCs are heavily dependent on glycolysis [80] to meet their energetic demand compared to neutrophil progenitors, which shifts their metabolism toward OXPHOS during differentiation. However, the reason for the initiation of the metabolic shift during differentiation, previously believed to be due to higher oxygen concentration, has been recently challenged. As discussed previously, the oxygen gradient in the bone marrow decreases in the endosteal region. Thus, there must be another factor other than oxygen availability influencing the metabolic shift. 

The main trigger for metabolic reprogramming during hematopoiesis, apart from differential oxygen availability in the bone marrow [11] is autophagy [95]. Autophagy will allow the metabolic shift toward FAO-OXPHOS by enabling lipid droplet breakdown, providing sufficiently free fatty acids [95]. The inhibition of autophagy-mediated lipid degradation or fatty acid oxidation, accompanied with a two-fold increase of the mitochondrial content, was shown to be sufficient to cause defective neutrophil differentiation. The highest autophagic activity was measured in the MB and MC stage, which indicates a more mitochondria-dependent metabolism during these stages [95]. However, the mechanism of how mature neutrophils modulate metabolic fluxes and switch their metabolism back to glycolysis remains to be discovered. 

### 4.2. Neutrophils’ Metabolic Shift from Plasma to Tissues

After their release from the bone marrow through sinusoidal capillaries, neutrophils enter the plasma fraction of the blood. Circulating neutrophils are considered quiescent and have a low transcriptional activity [96], which does not reflect their inability to modulate gene expression during infection or inflammation [96,97].

Like HSC, neutrophils are also highly dependent on HIF-1α regulation [3]. HIFs are transcription factors, recognized as key modulators to hypoxic stress. HIFs are heterodimers containing an oxygen-labile α cytosolic subunit and a more stable nuclear β subunit. In neutrophils, two HIF isoforms are known as HIF1α and HIF2α [3,98]. HIF1 is the major transcriptional regulator involved in the adaptation to low oxygen environments in terms of glycolytic enzyme expression upregulation. Similar to HIF1, HIF2 has an important role in neutrophils, but regulates a different set of genes [99]. If the main role of HIF1 is to facilitate a metabolic adaptation to a low oxygen environment, then HIF2 is mainly involved during the inflammation resolution, which regulates apoptosis signaling pathways [99]. Moreover, neutrophils accumulate antioxidants, such as ascorbate (vitamin C), capable of reducing available oxygen in cells, which limits ROS production and oxidative damage. Neutrophils contain high intracellular ascorbate concentrations (1–2 mM) compared to plasma ascorbate concentration (20–80 μM) and are known to increase their intracellular ascorbate intake even more during oxidative burst (10–20 mM), which contributes to their chemotaxis and ROS generation [100]. However, high ascorbate concentrations are known to promote HIF1α degradation, even at low (1–3%) oxygen concentrations [101,102]. This suggests that the neutrophil glycolytic phenotype is more likely linked to parameters, such as low oxygen, high glucose, and low energetic needs.

Upon inflammation or infection, neutrophils transmigrate into tissues (diapedesis) and will further evolve in various microenvironments where they will exert different functions. The metabolic requirements for different neutrophil functions is represented in Table 2. However, how neutrophil metabolism is modulated during this transition remains largely unexplored. The recruitment of neutrophils to inflammation sites is a multi-step process consisting of (i) a selectin-mediated rolling, (ii) a chemokine-induced activation, and (iii) an integrin-dependent strong adhesion followed by trans-endothelial migration (TEM).

For a long time, neutrophil migration was considered unidirectional and, together with the adhesion cascade, has been exhaustively characterized [103,104,105]. However, recently, Woodfin and colleagues discovered that neutrophils were able to retro-transmigrate back into the circulation and identified junctional adhesion molecule C (JAM-C) as the key regulator of directional TEM [106]. The retro-trans-endothelial migration (rTEM) was thought to contribute to the dissemination of systemic inflammation. The existence of rTEM also raises the question whether neutrophils returning from a different environment would exhibit phenotypic and metabolic changes and contribute to the heterogeneity found in the circulating neutrophilic pool in both healthy and nonhealthy subjects. The heterogeneity found among neutrophils was recently reviewed by Silvestre-Roig and colleagues [107].

Not much is known about the metabolic changes occurring during migration in vivo. However, using a zebrafish model, the function of mitochondria was shown to play a crucial role in the migration fitness of neutrophils into tissues. Zhou and colleagues showed that, by creating a mitochondrial DNA polymerase mutant, neutrophils had an altered motility in vivo [77]. It is well documented that, in many migrating cells, such as cancer cells and lymphocytes, motility is induced with localized ATP production (reviewed by Furnish and Caino, 2019; Ledderose et al., 2018 [108,109]). In neutrophils, it seems that it is the maintenance of the mitochondrial membrane potential that is crucial for migration [77], which suggests that mitochondria can drive cell migration with additional mechanisms, not only through ATP production.

Another report describes the role of exogenous glutamine in neutrophils chemotaxis. The authors showed that glutamine administration impairs neutrophils migration during endotoxemia, induced by *E. coli* lipopolysaccharide (LPS) [112]. The migration of neutrophils was enhanced in the absence of glutamine, which suggests a drop in glutamine concentration during infection would facilitate neutrophils migration to inflammation sites. In endothelial cells, glutamine was shown to promote proliferation and not migration [113]. In transformed breast cells, glutamine deprivation enhanced inflammatory gene expression [114]. Thus, the enhanced neutrophil motility could be explained by an active phenotype, which is promoted by glutamine deficiency causing metabolic stress. Thus, the administration of glutamine during infection should be wisely reviewed since it seems to have potentially deleterious anti-inflammatory properties.

The role of neutrophils in inflamed tissues differs depending on the cause of inflammation. In the case of infection, neutrophils will be the first line of defense, using a myriad of anti-bacterial mechanisms to overcome pathogen propagation. However, in the case of auto-immune diseases, such as systemic vasculitis, systemic lupus erythematosus, rheumatoid arthritis, and some cancer types, neutrophils acquire a pro-inflammatory phenotype, which induces tissue damage, cancer progression, and, thus, the severity of the disease. It is, therefore, important to consider a heterogeneity in terms of pro-inflammatory or anti-inflammatory phenotypes among neutrophil populations depending on the inflammation type/source and local micro-environment.

### 4.3. Neutrophils Metabolic Shift upon Neutrophil Antimicrobial Functions Activation

Several metabolic pathways have been shown to be required for neutrophil antimicrobial functions, as briefly reviewed below. However, the impact of antimicrobial function activation on a potential metabolic shift has not been reported so far and remains an important question to be addressed.

Borregaard and colleagues provided the first link between metabolism and antimicrobial activity [18]. The authors demonstrated that resting neutrophil produce ATP mainly through glycolysis from glucose taken up from the surrounding medium. During phagocytosis, no significant change of the ATP generation rate was reported, while a fall in intracellular ATP concentration was observed, due to energy utilization. Consistently, it was shown that mitochondria do not play a role in phagocytosis regulation [19]. Nevertheless, neutrophils were also shown to use different energy sources for different functions, demonstrating the versatile nature of neutrophils [115]. 

More recently, other anti-microbial functions, such as NADPH production via NOX and NET formation, were shown to be glucose-dependent and glycolysis-dependent [63,116]. The NADPH used by NOX originates from the PPP, which demonstrates the importance of a tightly regulated glucose metabolism in neutrophil functions. Moreover, hyperglycemia was shown to promote NET formation [14], explaining the increased number of spontaneous NETs observed in Type 2 diabetes mellitus patients. However, deficient NET formation has been reported in high glucose concentrations, describing the released NETs as unstable and containing decreased amounts of anti-microbial peptides compared to NETs released in physiological glucose concentrations [117,118]. It has been suggested that high glucose concentration activates neutrophils, and hinders them to react to additional stimuli, such as LPS [119,120]. It is currently well accepted that hyperglycemia impairs many neutrophils’ key functions [121], such as phagocytosis, ROS production, and bacterial killing, as reviewed by Insuela and colleagues [121]. The exact mechanism involved in neutrophil impairment in high-glucose conditions remains to be identified. Nevertheless, since glucose concentration seems to be the centerpiece of neutrophil functions, the choice of the cell culture medium for in vitro experiments is crucial (e.g., RPMI medium contains 11 mM glucose).

On the other hand, in the absence of extracellular glucose, ATP generation in neutrophils is exclusively associated with glycogenolysis, which consists of breaking down glycogen molecules present in the cytosol of neutrophils. It has been demonstrated that, in the absence of glucose, ATP generation decreases from 1.3 fmol/cell/min to 0.75 fmol/cell/min [18]. Yet, phagocytosis was shown to upregulate ATP generation (and glycogenolysis) up to 1.2 fmol/cell/min [18]. However, it has not been reported whether or not NET formation efficiency is modulated in these conditions, which raises the question of NET formation potential in glucose-poor environments. Moreover, the differences of glucose-associated anti-microbial functions in terms of efficiency and energy metabolism during glycogenolysis or in the presence of glucose remains to be further investigated.

The importance of glycogen storage and utilization has been elegantly demonstrated by Walmsley and colleagues. The authors showed that, in the absence of Phd2, the HIF-hydroxylase, by inhibiting the HIF pathway activation, neutrophil functions were enhanced [22]. Moreover, hypoxia pre-conditioning activating HIF and increasing glucose utilization, was shown to increase the efficiency of neutrophils in terms of antimicrobial activity [122]. Since HIF-1 plays a crucial role in the adaptation to low oxygen concentrations by upregulating glycolysis, we can appreciate that the regulation of the glycolytic flux and glycogen storage are important during pathogen clearance but also during inflammation resolution [22]. Until now, no direct link between glutaminolysis or a shift toward this metabolic pathway upon neutrophil antimicrobial function activation has been reported.

As previously mentioned, NET formation relies on glucose metabolism in terms of NOX activity (PPP) and energy production (glycolysis), which correlates with observation of Glut1 and Glut3 upregulation upon PMA stimulation [58]. However, besides NOX-dependent NETs (ND-NETs), NOX-independent NETs (NI-NETs) have also been characterized. NI-NETs are induced by calcium ionophores, which enhances mitochondrial ROS production [123] and are, therefore, believed to be less dependent on glucose metabolism. Although the source of ROS differs between ND-NETs and NI-NETs, the difference in terms of energy metabolism has not been investigated. Moreover, oxygen seems to be the centerpiece of NET formation. However, in inflammatory conditions, oxygen concentration tends to be very low compared to the atmospheric oxygen concentration (21%). This parameter should be taken into account when investigating ROS-induced anti-microbial functions in vitro. In addition, increased pH has also been described as an enhancer of NI-NETs [124,125]. The authors explained the pH sensitive nature of NI-NETs by demonstrating that many key enzymes participating in NI-NET formation have an alkaline pH optimum. However, the authors did not investigate changes in energy metabolism in increased pH conditions. It is, however, known and described in leukocytes that glycolysis is optimal at pH 7.5 and is enhanced in alkaline pH [126]. Overall, these reports clearly demonstrate the impact of environmental changes, such as pH on enhancing neutrophil key functions during inflammation. 

### 4.4. Metabolic Shift during Infection

Metabolic shifts may occur at infectious sites in response to changes in the neutrophil microenvironment due to the presence of pathogens, rather than a result of neutrophil activation, as illustrated by the depletion of oxygen due to bacterial aerobic respiration [39] in comparison with neutrophil ROS production [127]. However, the concentrations of glucose or glutamine at infectious sites remain largely unknown so far. Since most changes in metabolism are linked to alterations in the carbon and nitrogen energy metabolism, understanding these changes will enable a better characterization of neutrophil adaptation to pathophysiological conditions. Some examples of metabolic modulation during infection will be discussed afterward.

In general, intracellular bacteria trigger “host core defense mechanisms,” which consist of inducing the production of ROS and reactive nitrogen species. These core functions in host cells are controlled by NF-κB and are activated by pathogen-associated molecular patterns (PAMPs). Several PAMP-associated NF-κB targets are linked with metabolic reactions, such as heme oxygenase-1, Ca^2+^ transporters, divalent metal ions, adenosine- and adenosine-monophosphate deaminases, indolamine-2,3-dioxygenase, and upregulation of mitochondrial O_2_ respiration [128]. Besides NF-κB, another transcription factor, such as HIF-1, has also been linked to host defense mechanisms. Strikingly, the stabilization and activation of HIF-1 during infection was shown to be oxygen-independent [129], which raises the question of intracellular oxygen availability during intracellular pathogen infection. In neutrophils, several intracellular pathogens have been shown to induce a “pro-bacteria” metabolism. *Francisella tularensis*, which is the causative agent of tularemia, is able to inhibit neutrophil ROS production by secreting several acid phosphatases [130]. Similar acid phosphatases are produced by C*oxiella burnetii* and released via the T2SS, which causes a dramatic decrease in neutrophil NADPH oxidase and, thus, ROS production [131].

Besides modulating the intracellular compartment, many pathogens shape the extracellular infectious microenvironment. Together with glucose and other carbohydrate consumption, pathogens can also consume oxygen, which leads to a transition from inflammatory hypoxia to infectious hypoxia. Many oxygen-utilizing bacteria, such as *E. coli*, can utilize oxygen at nanomolar levels [132], which explains the severity of tissue hypoxia during infection. 

Infectious hypoxia was recently reviewed by Arena and colleagues [38]. In addition to resident host cells and infiltrating immune cells, bacteria also consume oxygen, which prevents its further use by neutrophil NADPH oxidase and illustrates the “battle for oxygen” occurring during bacterial infections.

How and if tissue oxygenation and glutamine or glucose availability during infection can modulate the energy metabolism of neutrophils and other immune cells remains to be studied. It seems possible that a metabolic shift will occur not only upon arrival to the infectious inflammation site but also during inflammation, where the microenvironment may change.

## 5. Importance of Metabolic Shifts in Neutrophil Population Heterogeneity

In recent years, several observations had led to the understanding that neutrophils do not always form a homogenous population, especially in auto-immune disease and cancer. The identification and classification of different neutrophil subtypes together with immunometabolism has opened a new field of research discussed hereafter.

### 5.1. Tumor-Associated Neutrophils 

Neutrophils are mostly associated with anti-tumoral functions, such as direct tumor cell killing and antigen presentation, which increases cytotoxic T lymphocyte-mediated anti-tumor immunity. However, it is now clear that neutrophils do not form a homogenous population and can play an important role in tumor progression by impairing the activation of CD8^+^ T cells and enhancing tumor invasion through NETosis [133,134]. Two major neutrophil subpopulations have been intensively studied in the tumor microenvironment, which include tumor associated neutrophils (TANs) and myeloid-derived suppressor cells (MDSCs). First, defined in 2007 [135], MDSC are the most studied neutrophil-like cell population in cancer progression, present in great numbers in several cancer models [136,137,138]. It was first suggested that MDSCs are immature myeloid cells, able to differentiate into macrophages (tumor-associated macrophages, TAMs) or neutrophils (tumor-associated neutrophils, TANs) based on their myeloid origin (PMN-MDSCs and M-MDSCs). Metabolically, MDSCs are described as flexible, able to sense and adapt to different TMEs. For example, several studies point out the importance of lipid oxidative metabolism in these cells, especially in low glucose availability [139,140,141]. However, a recent publication challenges the link between lipid uptake and an energetic switch in MDSCs. Even if an upregulation of fatty acid transport protein 2 in PMN-MDSCs was observed, it did not lead to changes in the energy metabolism of MDSCs [142]. Nevertheless, it did increase the synthesis of prostaglandin E_2_ from arachidonic acid_,_ contributing to the immunosuppressive activity of PMN-MDSCs [142].

Similar to macrophages, (M1 for anti-tumor and M2 for pro-tumor [5,6]) neutrophils are also reported to form two populations, N1 (anti-tumor/anti-inflammatory) and N2 (pro-tumor/pro-inflammatory) [7]. Even if phenotypically different, there is no current marker to distinguish N1/N2 neutrophils in the tumor micro-environment.

The pro-inflammatory/pro-tumor neutrophils (N2), which are also called N2 TANs, are characterized by the release of excessive ROS, which enable cancer progression in several ways. Since increased ROS stabilizes HIF1, it promotes VEGF and MIF production, which are both important in cancer progression and chemotherapy resistance [143].

Because N2 TANs promote cancer metastasis to distant organs, they are now considered a potential therapeutic target. Currently, not much is known about the energy metabolism of TANs. However, similarities with tumor-associated macrophages (TAMs) can be drawn.

In the tumor microenvironment, high numbers of M2 TAMs (pro-tumor/pro-inflammatory phenotype) is associated with tumor growth, metastasis, angiogenesis, and poor prognosis. It is progressively acknowledged that, because of their high plasticity, macrophages undergo metabolic changes that establish their functional fate. The metabolic shift toward the M2 phenotype in TAMs occurs when they accumulate in hypoxic areas of the tumor micro-environment [144]. In these conditions, TAMs will be exposed to lactic acid produced by the cancer cells, stabilizing HIF-1α, even in the presence of increased oxygen levels [145]. Consequently, M2 TAMs will adapt to aerobic glycolysis, which is also known as the Warburg effect, a hallmark of cancer.

Recent evidence suggests that not only do cancer cells modulate the metabolism of immune cells, but also immune cells, such as macrophages, enhance tumor hypoxia by depleting oxygen and secreting TNFα by inducing the glycolytic phenotype observed in tumors [146].

Although similarities have been drawn between PMN-MDSCs and N2 TANs, recent findings suggest otherwise. Results from transcriptomic analyses comparing MDSCs, TANs, and normal neutrophils revealed that MDSCs resemble more normal neutrophils, than TANs [147], which indicates that MDSCs and TANs are clearly two different populations with different mechanisms. Compared to MDCSs, TANs showed a lower expression of granule proteins and the NOX complex, which are both important for major neutrophil functions [147]. Recently, a new tumor-associated neutrophil population was described by Rice et al., the c-Kit+ tumor-elicited neutrophils (TENs) [148]. This subpopulation was characterized with the ability to use mitochondrial oxidative metabolism in low glucose availability. The authors showed that, in a limited glucose supply, TENs were able to use FAO-OxPHOS metabolism to maintain their NADPH supply, which is essential for ROS production. 

Another recent publication highlights the link between neutrophil mitochondria and motility in vivo [75]. The authors showed that, similar to cancer cells, migrating neutrophils have mitochondria localized in the front and in the rear, which could serve the purpose of localized ATP production. Bao and colleagues, however, demonstrated that, although neutrophils produce most of their ATP via glycolysis, mitochondria are essential for producing the ATP that triggers their activation via a purinergic signaling process [111]. They observed that external stimulation of neutrophils with fMPL increased the ∆Ψ_m_ and released ATP, which leads to Ca^2+^ mobilization and oxidative burst. 

### 5.2. Low-Density Neutrophils

Low-density neutrophils (LDNs) are a population of neutrophils obtained from the PBMC (peripheral blood mononuclear cells) fraction after density gradient centrifugation. LDNs are currently divided into two subpopulations, which include the immunosuppressive LDNs, mainly found in cancer, pregnancy, infections, and systemic inflammation; and the proinflammatory LDNs, mainly found in autoimmune diseases, such as systemic lupus erythematosus and anti-neutrophil cytoplasmic autoantibody (ANCA) vascularitis, and often referred to as low density granulocytes (LDGs). 

Although LDNs have been found in many tumor microenvironments, their presence in liver metastasis seems to be the most relevant [149]. Recently, Hsu et al. showed that cancer-cell-produced granulocyte colony-stimulating factor (G-CSF) was involved in the mobilization of immature low-density neutrophils (iLDNs), which promote extensive liver metastasis. These immature cells constitute another subpopulation of LDNs found in several disease models. Mature LDNs, however, seemed to inhibit the formation of liver metastases. In liver metastases, iLDNs were shown to engaged in mitochondrial-dependent ATP production and were able to perform NETosis under nutrient-deprived conditions (without glucose). This is uncommon since NETosis has been reported to rely strongly on glucose availability in mature neutrophils [116]. The authors reported that iLDNs relied on the catabolism of glutamate and proline to support mitochondrial-dependent metabolism in the absence of glucose.

With some exception discussed previously, not much is known about tumor/metastasis-promoting neutrophils. Since similarities are drawn between TAMs and neutrophil-like tumor promoting cells (PMN-MDCSs, TANs, TENs, LDNs), the question regarding if neutrophils serve a similar purpose in the tumor microenvironment remains uncertain.

Taken together, it seems like tumor-promoting neutrophils are metabolically more flexible than circulating neutrophils, which enables them to adapt in situ to different tumor micro-environments.

## 6. Conclusions

Neutrophil metabolism plays a central role in their survival in changing environments encountered during their life cycle beginning from their development to the activation of their antimicrobial functions. Neutrophil metabolism dysregulation has been observed in many inflammatory diseases such as diabetes, sepsis, cystic fibrosis, lupus, or atherosclerosis [150]. The link between metabolism modulation and several activation pathways has been established in many reports. However, no direct link has been established with changes of their micro-environment and the availability of key metabolites such as glucose, glutamine, and oxygen. Major efforts should be made in the future to assess local micro-environmental changes, which requires the development of new and non-disruptive methods to perform quantification in situ. As outlined in Figure 1 and Table 1, knowledge remains scarce in pathophysiological conditions. It has to be highlighted in this section that, without these data, any attempt to validate the relevance of neutrophil metabolic adaptation in health and diseases will be challenging.

Most of the recent studies are aimed at describing the impact of neutrophil metabolic shifts on neutrophil survival and activation. However, the impact of neutrophil activation on neutrophil metabolic shift induction should also be considered, even though it remains largely unexplored. This idea may be supported by the fact that neutrophil metabolic activity and activation may differentially “imprint” their microenvironment (oxygen availability, pH, glucose, or glutamine concentration), potentially leading to secondary metabolic shifts. As a consequence, the contribution of neutrophil metabolic shifts in the neutrophil lifecycle has likely been underestimated so far and, thus, represents an attractive emerging field of research. It is anticipated that neutrophil metabolic shifts will be different in inflammatory and infectious diseases and may be considered in the future as a specific “signature” for the development of the pathology. Further investigations are urgently needed to fully understand how neutrophils adapt to their microenvironment and to decipher to which extent their metabolic shifts impact the outcome of inflammatory diseases, to envision new therapy strategies.

## Figures and Tables

**Table 1:** Oxygen, glucose and glutamine concentrations in the bone marrow, plasma and tissues under physiological conditions (upper panel) and pathophysiological conditions (lower panel).

**Physiological Conditions**			
**Compartment**	**Oxygen [c]**	**Glucose [c]**	**Glutamine [c]**
Bone marrow	1.3–2.9%	?	?
Plasma	0.9%	5 mM	0.5 mM
Tissue	1–11%	?	2–20 mM
**Pathophysiological Conditions**			
**Compartment**	**Oxygen [c]**	**Glucose [c]**	**Glutamine [c]**
Bone marrow	?	?	?
Plasma	?	?	↓
Tissue	↓	↓	↓

**Table 2:** Metabolic pathways involved in neutrophil functions.

Neutrophil Function	Metabolic Requirements	References
Phagocytosis	Glycolysis	[18]
ROS production (NOX)	PPP, Glutaminolysis	[63,72]
Degranulation	Glycolysis	[12,110]
NET formation	PPP, Glycolysis	[14,63]
Chemotaxis/migration	Glycolysis, mitochondrial metabolism	[76,77,111]

ROS—reactive oxygen species; NOX—NADPH oxidase; NET—neutrophil extracellular trap; PPP—pentose phosphate pathway.
